# Incidence and time course of new contrast-enhancing lesions on MRI after proton versus photon radiotherapy in glioma patients

**DOI:** 10.1007/s00234-025-03829-1

**Published:** 2025-11-24

**Authors:** Arne Grey, Thiem Justus, Hannes Wahl, Kay Engellandt, Annekatrin Seidlitz, Rebecca Bütof, Mechthild Krause, Enrico Michler, Matthias Meinhardt, Esther G. C. Troost, Jennifer Linn

**Affiliations:** 1 Institute of Neuroradiology, Faculty of Medicine and University Hospital Carl Gustav Carus, Technische Universität Dresden, Dresden, Germany; 2 Department of Radiotherapy and Radiation Oncology, Faculty of Medicine and University Hospital Carl Gustav Carus, Technische Universität Dresden, Dresden, Germany; 3 OncoRay - National Center for Radiation Research in Oncology, Faculty of Medicine and University Hospital Carl Gustav Carus, Technische Universität Dresden, Helmholtz-Zentrum Dresden-Rossendorf, Dresden, Germany; 4 Department of Nuclear Medicine, Faculty of Medicine and University Hospital Carl Gustav Carus, Technische Universität Dresden, Dresden, Germany; 5 Institute of Pathology, Faculty of Medicine and University Hospital Carl Gustav Carus, Technische Universität Dresden, Dresden, Germany; 6 National Center for Tumor Diseases (NCT), Partner Site Dresden, Dresden, Germany

**Keywords:** Proton therapy, Photon therapy, Radiotherapy, Glioma, Radiation necrosis, Contrast-enhancing lesions

## Abstract

**Purpose:**

Our aim was to evaluate the incidence and time course of new contrast-enhancing lesions (CEL) after high-dose irradiation therapy (RT) with protons or photons in adult glioma patients.

**Methods:**

We retrospectively analyzed the MRI data sets of 240 consecutive, adult patients with gliomas who received high-dose photon (*n* = 141) or proton (*n* = 99) therapy. All available follow-up MRIs were analyzed for incidence of any new CELs. Postradiogenic contrast-enhancing lesions (pCEL) were distinguished from tumor progression either histologically (definite pCEL) or based on follow-up imaging demonstrating spontaneous complete or partial regression (probable pCEL).

**Results:**

During a median follow-up period of 30 months, we observed any new CEL in 74.2% of patients. Of these, 53.9% met the criteria for definite or probable tumor progression, while 28.8% met the criteria for definite or probable pCEL. The incidence of pCEL was significantly higher after proton therapy (47.5%, *n* = 47 of 99 patients) versus photon therapy (15.6%, *n* = 22 of 141 patients, *p* < 0.0001). The median time interval between end of RT and first evidence of pCEL was significantly shorter after proton therapy (11.4 months) compared to photon therapy (16.5 months, *p* = 0.035).

**Conclusion:**

Postradiogenic CELs are a frequent finding in adult patients with gliomas, particularly in those treated with proton therapy. Awareness of the high incidence, variable morphology and time course of these treatment related changes is essential for correct interpretation of follow-up imaging findings and for appropriate patient management.

**Supplementary Information:**

The online version contains supplementary material available at 10.1007/s00234-025-03829-1.

## Introduction

Radiotherapy (RT) with photons is an essential part of the adjuvant therapy in primary brain tumor patients [1]. Due to the increasing availability of proton therapy in Europe and worldwide, and due to the growing body of evidence regarding the benefits of this treatment modality, proton beam therapy is a potential alternative to photon therapy [2]. The physical properties of proton therapy, i.e. the Bragg peak, offer a highly conformal dose delivery to the target volume, while sparing the surrounding tissue and organs at risk due to the steep dose gradient and the reduced integral biological dose [3, 4]. These properties make proton therapy highly attractive for the treatment of pediatric or adult primary brain tumor patients [5].

Since the target volume consists of the tumor bed (and residual gross tumor) enlarged by a margin, covering microscopic tumor spread, and since external beam deposits additional low radiation doses outside the target volume, RT may be associated with different types of radiation-induced brain injury, including leukoencephalopathy, brain atrophy, vascular lesions, calcifications and/or contrast-enhancing lesions (CEL) [6].

Depending on their onset after therapy, radiation-induced brain injuries can be classified into acute, early-delayed and late-delayed changes [6]. Postradiogenic CEL occur months to years after RT, i.e. constitute early-delayed or late- delayed postradiogenic changes. CEL is an umbrella term for a broad variety of lesion types with different clinical significance and different underlying pathomechanisms, which are not yet fully understood [6–8]. Their morphological spectrum ranges from dot-like or speckled, often asymptomatic lesions (Fig. 3) to large, symptomatic tumor-like lesions with central necrosis (i.e. radiation necrosis; Figs. 2 and 4) [6]. The periventricular region seems to be particularly prone to postradiogenic injury, which might be explained by presence of neural stem cells with an increased radiosensitivity [6].

For photon therapy, the reported incidence of pCELs varies from 5 to 30% in the literature, depending on the diagnostic criteria and method used for its identification as well as on tumor specific and treatment specific factors [9–14]. With regard to proton therapy, available data predominately focuses on pediatric cases and/or on low grade glioma [10, 12, 15–18].

In this study we aimed to compare the incidence and timing of new or progressive pCELs after photon versus proton therapy in a large cohort of adult glioma patients.

## Materials and methods

### Patient population

We used our electronic, in-hospital database of radiotherapy records to identify all adult patients who underwent intracranial high dose RT with 54–60 Gy with protons or photons between 01/2012 and 03/2019 to ensure an adequate follow-up interval. Only glioma patients in good to moderate general clinical condition (ECOC 0–2) were regarded suitable for – either proton and photon - high-dose RT. After clinical introduction of proton-beam therapy at our institution, this alternative therapeutic option was offered in all cases were reimbursement by the medical assurance agencies was guaranteed based on selective contracts. The definite choice of treatment modality depended on the treating physician´s and the patient´s decision. There was no systematic assignment of patients to one of the therapeutic options based on clinical patient or tumor characteristics. A small subset of cases from this cohort has been included in a previous publication [19].

**Fig. 1:**
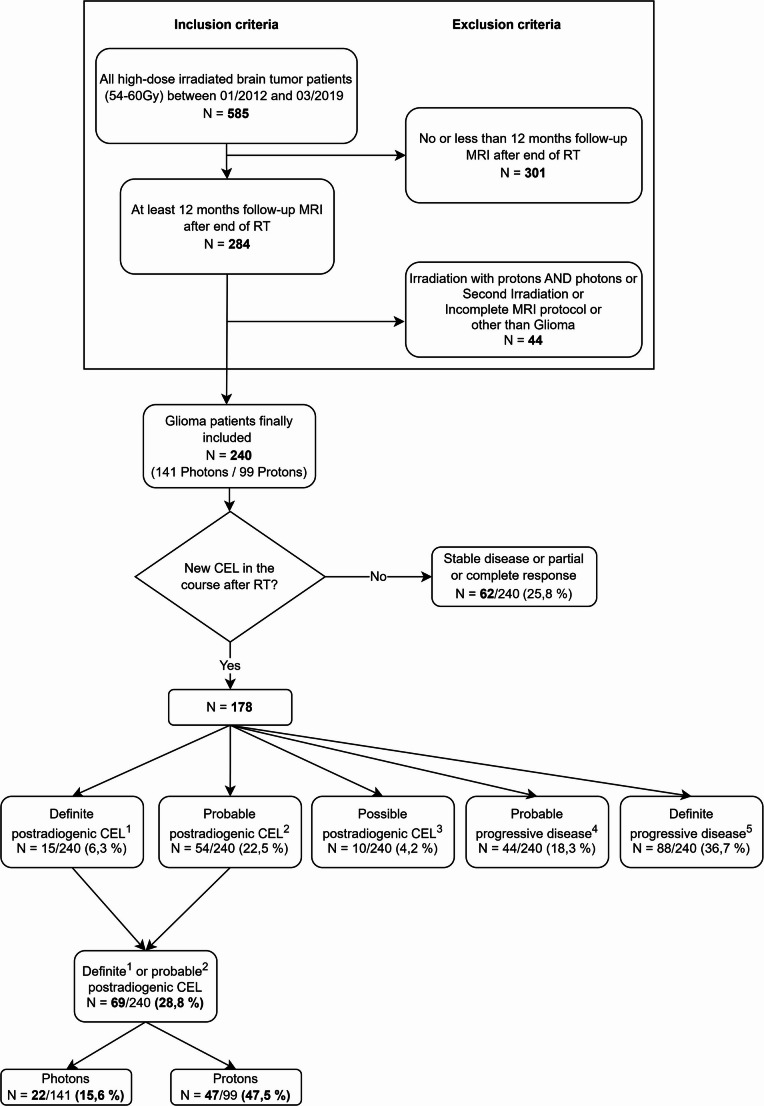
Flow chart illustrating the retrospective patient identification and their assignment to the different patient groups. Gy: Gray; N: absolute number; MRI: magnetic resonance imaging; RT: radiation therapy; CEL: contrast enhancing lesions; Photons: photon therapy; Protons: proton therapy

Patients were included in this retrospective analysis if they met the following additional inclusion and exclusion criteria (Fig. 1):

Inclusion criteria:

- neurosurgical tumor resection performed prior to RT.
- histological diagnosis of a glioma.
- MRI performed immediately after surgery and prior to start of RT available for analysis.
- Follow-up MRIs including at least a T2-weighted, a diffusion-weighted and a T1-weighted sequence prior and after administration of intravenous contrast agent available for a minimum of 12 months after RT.

Exclusion criteria:

- History of previous RT.
- combined RT with protons and photons.
- MRI imaging quality not suitable for interpretation, e.g. due to severe motion artifacts or lack of essential sequences as mentioned above.

Demographic and clinical patient data were obtained from our institutional electronical data base of medical records.

### Tumors characteristics

Tumor diagnosis (histology and WHO grading) was based on the 4th edition of the WHO classification for patients treated between 2012 and 2016 [20], and on the updated 4th edition [21] for cases treated from 2016 onwards as extracted from the medical records (Table 1).

**Table 1:** Patient and tumor characteristics for the entire study population, the photon therapy and the proton therapy patient group

Patient characteristics		Entire population (%)	Photon therapy (%)	Proton therapy (%)	*p*-value (Photon vs. Proton)
n		240 (100)	141 (58.8)	99 (41.3)	
Age (median years)		51 (IQR 39–62)	53 (IQR 44–63)	48 (IQR 35–59)	Kruskal-Wallis: *p* < 0.001
Gender	male	135 (56.2)	80 (56.7)	55 (55.6)	Chi-Square: *p* > 0.05
	female	105 (43.8)	61 (43.3)	44 (44.4)	
WHO grade (at initial diagnosis*)	I	3 (1.2)	1 (0.7)	2 (2)	Chi-Square: *p* < 0.01
	II	22 (9.2)	7 (5)	15 (15.2)	
	III	83 (34.6)	41 (29.1)	42 (42.4)	
	IV	132 (55)	92 (65.2)	40 (40.4)	
	**LGG** (WHO I or II)	25 (10.4)	8 (5.7)	17 (17.2)	Chi-Square: *p* < 0.05
	**HGG** (WHO III or IV)	215 (89.6)	133 (94.3)	82 (82.8)	
WHO grade (re-defined according to WHO classification 2021**)	**LGG** (WHO 1 or 2)	22 (9.2)	6 (4.3)	16 (16.2)	Chi-Square: *p* < 0.05
	**HGG** (WHO 3 or 4)	218 (90.8)	135 (95.7)	83 (83.8)	
	HGG IDH-wildtype	124 (56.9)	81 (60)	43 (51.8)	Chi-Square: *p* < 0.05
	HGG IDH-mutant	71 (32.6)	31 (23)	40 (48.2)	
	HGG IDH not available	23 (10.6)	23 (17)	0 (0)	
Histology (at initial diagnosis*)	Glioblastoma	131 (54.6)	92 (65.2)	39 (39.4)	
	Astrocytoma	60 (25)	25 (17.7)	35 (35.4)	
	Oligodendroglioma	20 (8.3)	4 (2.8)	16 (16.2)	
	Oligoastrocytoma	23 (9.6)	18 (12.8)	5 (5.1)	
	Others	6 (2.5)	2 (1.4)	4 (4)	
Histology (re-defined according to WHO classification 2021 ***)	Glioblastoma	144 (0.6)	101 (71.6)	43 (43.4)	
	Diffuse Astrocytoma	58 (24.2)	23 (16.3)	35 (35.4)	
	Oligodendroglioma	32 (13.3)	15 (10.6)	17 (17.2)	
	Others	6 (2.5)	2 (1.4)	4 (4)	
CTx	no CTx or only neoadjuvant CTx	77 (32.1)	46 (32.6)	31 (31.3)	
	Concomitant **and/or** adjuvant CTX	163 (67.9)	95 (67.4)	68 (68.7)	
	adjuvant CTx only	55 (22.9)	19 (13.5)	36 (36.4)	
	concomitant +/- adjuvant CTx	108 (45)	76 (53.9)	32 (32.3)	

*n* absolute number, *IQR* interquartile range, *LGG* low grade glioma, *HGG* high grade glioma, *IDH* Isocitratdehydrogenase, *CTx* chemotherapy

* based on WHO classification for 2007 or 2016 [20, 21].

** based on WHO classification for 2021 [22] for cases with available immunohistochemistry and pathomolecular information.

*** Most likely diagnosis based on available histology, immunohistochemistry and molecular pathology for cases with available information.

For further analysis in the context of this study, a re-grading according to the current 5th edition of the WHO classification, introduced in 2021 [22], and an assessment of IDH-status were retrospectively made for those cases, in which the necessary immunohistochemical and pathomolecular information was provided in the original neuropathological reports (Table 1).

### Radiation treatment planning and delivery

As defined in the inclusions criteria, all patients eligible for this analysis had undergone a tumor resection prior to adjuvant RT (in combination with chemotherapy, depending on the tumor grade and MGMT promotor methylation status, see Table 1). The individual treatment regime was determined in the multidisciplinary neuro-oncological tumor board. For radiation treatment planning, computed tomography (CT) scans were obtained in supine position using an individual thermoplastic head immobilization system. Tumor bed volume (TBV) and potential residual tumor (gross tumor volume; GTV) were contoured on the CTs co-registered to the baseline MRI scans [T1-weighted (T1w), T2-weighted and contrast enhanced T1w images]. The clinical target volume (CTV) was generated by applying a 1–2 cm isotropic margin around the TBV, which was subsequently corrected for anatomical boundaries. In case of proton therapy, the dose to the CTV was planned using a robust optimization, considering the uncertainties in patient positioning and proton range, calculated individually depending on the used energy. For photon beam irradiation, a planning target volume (PTV) with an isotropic margin from CTV of 0.5 cm was created. 

The prescribed total proton and photon dose to CTV and PTV, respectively, was 54–60 Gy (relative biological effectiveness, RBE) and was delivered in 2 Gy (RBE)-fractions, corrected for the relative biological effectiveness of protons by a factor of 1.1. Protons with 230 MeV maximum beam energy were delivered with the double-scattering technique planned in XiO (Version V5.00.02, Elekta AB, Stockholm, Sweden) with field-shaping using lateral apertures and range compensators (2014–2017) or with the pencil-beam scanning technique planned on RayStation (Version 6.0, RaySearch, Stockholm, Sweden) with uniform single-field dose optimization (from 2018 onwards). Photon therapy was applied as 3D-conformal or step-and-shoot Intensity Modulated Radiation Therapy.

### MRI examinations

The following MRI data sets were analyzed: (1) postoperative MRI performed within 72 h after neurosurgical tumor resection, (2) MRI performed immediately prior to RT (baseline scan), (3) all available follow-up MRI scans. The first follow-up MRI was acquired approximately three months after the end of RT. All further follow up examinations were performed within three to six monthly intervals thereafter. Postoperative and baseline MRI immediately prior to RT were performed on 3 Tesla MR scanners, follow-up MRIs were acquired on 1.5T or 3 T scanners.

All MRI data sets included at least a T2w-or FLAIR-weighted-sequence, a diffusion-weighted sequence as well as a 2D or 3D T1-weighted sequence prior and after intravenous administration of a gadolinium-based contrast agent.

### PET-MRI examinations

In a subset of patients with new or progressive CELs, 11 C-methionine (MET) PET/MRI had been performed as part of the clinical work-flow to differentiate pCEL from tumor progression. If available, 11 C-MET-PET/MRI results were compared with MRI findings and histological diagnosis.

### MR imaging analysis

All MR data sets were analyzed by a board-certified neuroradiologist and questionable cases were additionally discussed with a senior board-certified neuroradiologist. Both readers were blinded with regard to clinical patient characteristics and irradiation technique used. The MRI scan performed immediately prior to RT was regarded as baseline.

All available follow-up MRIs were analyzed with respect to incidence of at least one new or progressive contrast-enhancing lesion (CEL) on contrast-enhanced T1-weighted sequences.

Date of first evidence of a new or progressive CEL was noted and the time interval between end of RT and detection of the respective lesion was determined.

### Histological findings in CEL

If histological tissue examination of a new or progressive CEL was available, the histological diagnosis (i.e. posttherapeutic changes, vital tumor tissue or both) was regarded as gold standard for its etiology (see below).

### Definition of CEL etiology

We defined the following criteria for etiological categorization of new or progressive CELs:

### Definite postradiogenic CELs

Diagnosis of ***definite*** postradiogenic CEL (definite pCEL) required available histological tissue examination demonstrating postradiogenic tissue injury.

#### Probable postradiogenic CELs

A new or initially progressive CEL was regarded as ***probable*** postradiogenic CEL (probable pCEL), if it fulfilled the following criteria:

- Spontaneous complete or partial regression of the CEL without a change of therapy within the available follow-up period, **and**.
- Absence of diffusion restriction in the respective localization on prior MRI (to exclude ischemic infarction as the underlying cause of the contrast-enhancement).

#### Possible postradiogenic CELs

A new or initially progressive CEL was regarded as ***possible*** postradiogenic CEL (possible pCEL), if it fulfilled the following criteria:

- Stable presentation or further progression of the CEL within the whole available follow-up period,
- Subependymal/periventricular localization, and/or.
- Presentation as dot-like parenchymal enhancement, and.
- Absence of diffusion restriction in the respective localization on prior MRI (to exclude ischemic infarction as the underlying cause of the contrast-enhancement).

#### Definite tumor progression-related CEL

Diagnosis of ***definite*** tumor progression-related CEL required histological tissue examination demonstrating vital tumor cells.

#### Probable tumor progression-related CEL

New or progressive CEL not fulfilling the criteria for definite, probable or possible pCEL without available tissue examination were regarded as ***probable*** tumor progression-related CEL, i.e. as progressive disease.

For all categories, additional CELs in other localization, which fulfilled the criteria of another of the above-mentioned categories were allowed and categorized accordingly, i.e. pCEL and tumor progression-related CEL might have been found in the same patient.

### Statistical analysis

R (4.2.1) was used for statistics.

For statistical analysis, LGG and HGG group assignment based on the initial diagnosis according to the WHO 2016 classification was used. Incidence of any new or progressive CEL, possible, probable and definite pCEL as well as probable and definite progressive disease was calculated for both therapeutic groups. Cases with histological and/or imaging findings indicating both postradiogenic as well as tumor-related CELs in the same patient were counted in both categories.

Cumulative incidences of (1) definite and probable pCEL and (2) definite and probable tumor-related CEL were compared between photon and proton groups and – for cases with available IDH-status - between IDH-mutant und IDH-wildtype HGG using Chi Square test.

Incidence of definite or probable pCEL in patients with concomitant and/or adjuvant and in those without or only neoadjuvant was assessed and compared for the whole cohort as well as for both therapeutic groups, separately. Within the proton cohort, we also compared the incidence of definite or probable pCEL in patients who received dual-scattering (DS) versus pencil-beam scanning (PBS) technique.

Median time interval from end of RT and first evidence of new or progressive postradiogenic or tumor-related CELs was calculated and compared between the photon and proton cohort using Wilcoxon tests.

Significance level was set to *p* ≤ 0.05.

## Results

### Study population, histological tumor diagnoses and radiation therapy

In total, *n* = 585 adult patients underwent intracranial high dose RT with 54–60 Gy with protons or photons between 01/2012 and 03/2019 at our institution. 240 of these patients (median age: 51 years, *n* = 135 males, 56.3%) fulfilled the additional inclusion and exclusion criteria and were further analyzed (for details see Fig. 1).

Initial histologic tumor diagnoses and WHO grading of the final study population as well as results of re-grading based on its 5th edition are shown in Table 1. The clinical diagnosis based on the 4th edition of the WHO classification was LGG in 10.4% (*n* = 25) and HGG in 89.6% (*n* = 215) of the whole population. 141 patients (58.8%; median age: 53 years) were treated with photons, while 99 patients (41.3%; median age: 48 years) received proton RT. The photon group comprised 5.7% LGG, and 94.3% HGG, while the proton group included 17.2% (*n* = 17) of LGG and 82.6% (*n* = 82) of HGG patients (Table 1).

Regarding the assignment to the LGG versus the HGG group, re-grading according to the latest WHO 2021 classification resulted in an upgrade from LGG to HGG in three cases, compared to the older WHO classification (Table 1). Information on IDH status was available in 217 of 240 cases (90.4%). Based on the 2021 WHO classification, our cohort consisted of 90.8% of high-grade glioma with IDH wildtype glioblastoma constituting the majority of cases (56.9% of the HGG group and 51,7% of the whole cohort) (Table 1). The proton patient group comprised a higher percentage of IDH mutant HGG patients than the proton group did (supplementary table 2a).

163 patients (67.9%, 95 with photons, 68 with protons) received concomitant and/or adjuvant chemotherapy (Table 1).

### Imaging findings

The median MR imaging follow-up was 30.4 months (range: 18.9 to 47.9 months). Additional to standard MRI, 55 patients had a methionine PET/MRI.

### Incidence of new CEL after RT

We observed any new CEL on follow-up after RT in 178 of 240 patients (74.2%), 102 of whom received photon therapy (102/178, 57.3%), while 76 patients were treated with proton therapy (76/178, 42.7%).

### Incidence of postradiogenic CEL

In total, *n* = 79 (79/240 = 32.9%) of all cases fulfilled the criteria of definite, probable or possible pCEL during follow up, among those were 22 patients with photon therapy (22/141 photon patients = 15.6%) and 56 patients with proton therapy (56/99 proton patients = 56.6%) (Table 2).

**Fig. 2:**
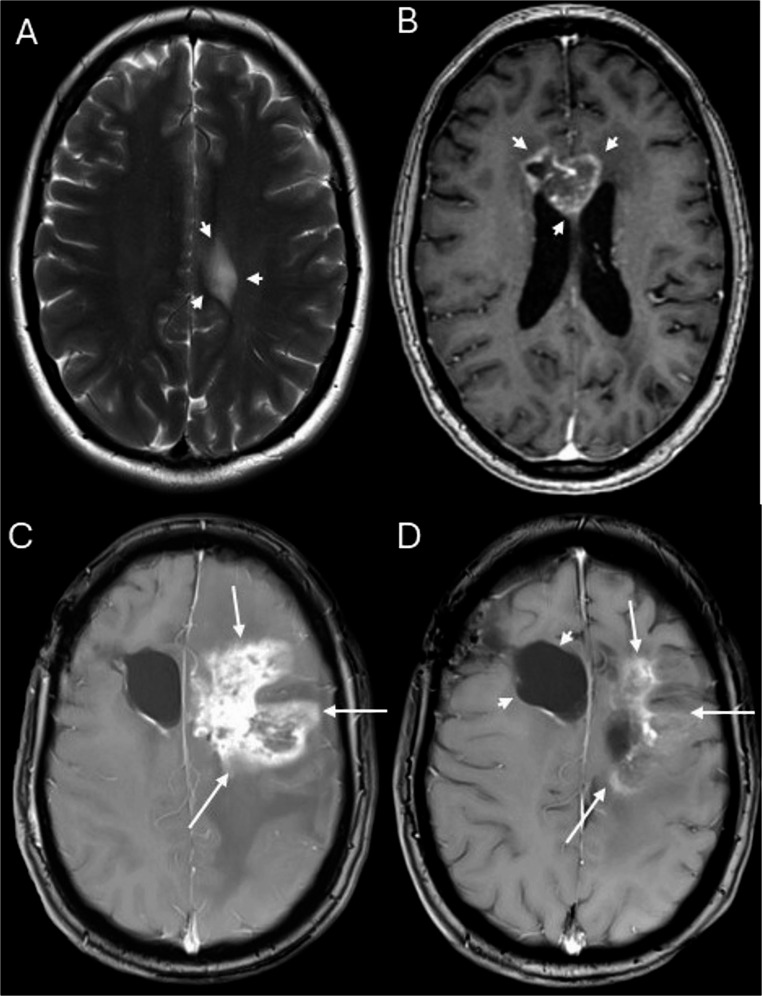
Extensive definite postradiogenic contrast-enhancing lesion (pCEL) after proton therapy. 39-year-old patient with non-enhancing astrocytoma WHO grade II in the left gyrus cinguli (**A**). Follow up MRI performed 31 months after radiation therapy with protons (**B**) shows a new contrast-enhancing lesion with rim enhancement and central necrosis (“radionecrosis”) in the corpus callosum (arrows in **B**). Histological tissue examination after resection of the lesion revealed necrosis and posttherapeutic tissue changes but no vital tumor tissue. Follow up MRI 15 months later (**C**) demonstrates extensive pCEL in the left frontal white matter (arrows in **C**) with significant perifocal oedema. Another follow-up MRI, performed 7 months later (**D**). shows partial regression of the pCEL without change of therapy (arrows in **D**) The arrow heads in **D** indicate the postsurgical defect after resection of the initial necrosis. **A**: T2-weighted sequence **B:**Axial reconstructions of the 3D-T1-weighted sequence after intravenous contrast application. **C, D**: Axial 2D-T1-weighted sequences after intravenous contrast application

**Fig. 3:**
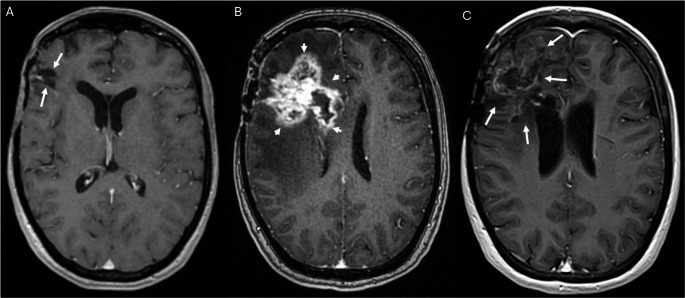
Example of a definite postradiogenic contrast-enhancing lesion (pCEL) after photon therapy. 33-year-old female patient with a glioblastoma. (**A**): Postoperative follow-up MRI after right frontal tumor resection shows surgical defect (arrows in **A**). Follow up MRI performed 26 months after radiation therapy with photons (**B**) shows a new, extensive contrast-enhancing lesion with central necrosis (“radionecrosis”) in the right frontal lobe including the corpus callosum (arrow heads in **B**). Histological tissue examination after partial resection of the lesion revealed necrosis and posttherapeutic tissue changes but no vital tumor tissue. Follow up MRI performed 43 months later (**C**) shows a residual state with significant regression of the pCEL and a parenchymal defect with gliosis (arrows in **C**). **A-C**: Axial reconstructions of the 3D-T1-weighted sequence after intravenous contrast application

**Fig. 4:**
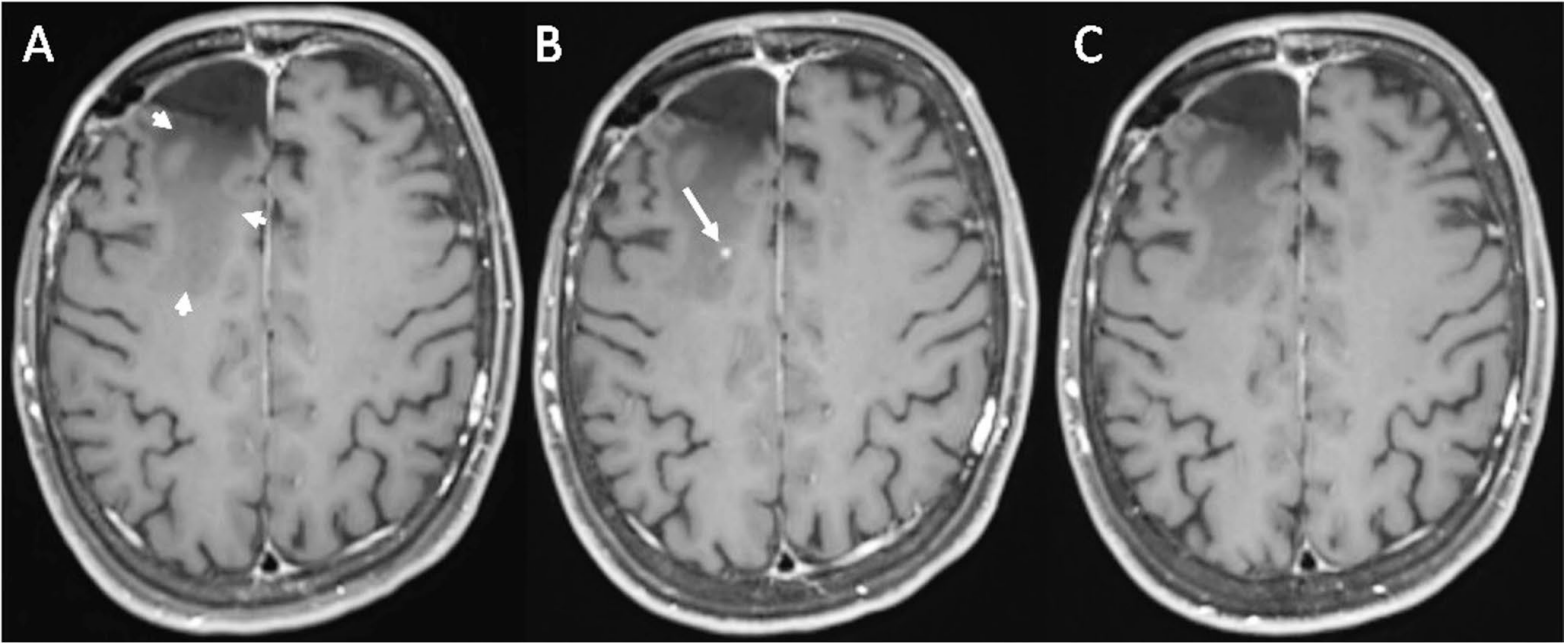
Example of a probable postradiogenic contrast enhancing lesion (pCEL)**.** 49-year old female patient with right frontal oligodendroglioma WHO grade III, who received tumor resection followed by proton radiotherapy. Follow up MRI performed 2years after end of radiation therapy (**A**) shows post-therapeutic leucencephalopathy (arrow heads) in the right superior frontal gyrus. Next available follow up MRI, performed 5 month later (**B**) depicts a new, dot-like parenchymal contrast enhancement (arrow). This lesion was present on two further follow-ups (not shown), but was no longer depictable on MRI performed 12 months after its initial detection (**C**), and therefore fullfilled the imaging criteria of a probable postradiogenic contrast-enhancing lesion. **A-C:**Axial reconstructions of the 3D-T1-weighted sequence after intravenous contrast application

Definite pCEL with histological evidence of radiation-induced injury (Figs. 2 and 3) were present in 15 of 240 patients, i.e. in 6.3% of the total study population and in 8.4% of cases with any new or progressive CEL. In 5 of these patients, tissue examination revealed also vital tumor tissue in addition to the postradiogenic changes. Two of these 5 patients fulfilled the imaging criteria for probable progressive disease in other localization. Criteria for probable pCEL (Fig. 4) were fulfilled in 54 patients, i.e. in 22.5% of the whole study population and in 30.3% of cases with any new or progressive CEL. These include 20 patients with additional imaging findings indicative of tumor progression at other localizations. In 21 of the 54 patients with probable pCEL, a methionine PET-MRI in addition to standard MRI was performed without evidence of increased amino acid metabolism, supporting the MR-based categorization as pCEL. Based on the patient files, we assessed the course of clinical symptoms for probable or definite pCEL (*n* = 69). Most pCELs were asymptomatic or mildly symptomatic, 18/69 patients (26%) showed severe symptoms that required treatment with either dexamethasone (15/69 patients) and/or bevacizumab (5/69 patients) .

Incidence of either definite or probable pCEL was significantly higher after proton therapy (47.5%) versus photon therapy (15.6%) for both the whole study population (47.5%, *n* = 47 of 99 patients, versus 15.6%, *n* = 22 of 141 patients, *p* < 0.0001) as well as for the subgroup of patients with high grade glioma (47.6%, *n* = 39 of 82 patients, versus 15%, *n* = 20 of 133 patients, *P* < 0.0001). For HGG cases with available IDH status (*n* = 195, 89.4%), there was no significant difference in the incidence of definite or probable pCEL between IDH-mutated (33.8%, *n* = 24) and IDH-wildtype cases (21.8%, *n* = 27; Chi²*p* = 0.066) neither for the whole population, nor for the photon or proton treatment groups (supplementary table 2b).

Within the small subgroup of low grade glioma, the incidence of definite or probable pCEL did not differ significantly after proton therapy (47.1%, *n* = 8/17 patients) compared to photon therapy (25%, 2/8 patients) (*p* = 0.294).

In the proton therapy group, incidence of postradiogenic CEL did not differ significantly between patients who received DS (*n* = 46; 58%) and PBS (*n* = 9; 40.9%) technique.

Incidence of definite or probable pCEL in the whole population and in the proton group was similar in patients who received concomitant and/or adjuvant CTx compared to those without (whole population: 31.3% versus 23.4% Chi² *p* = 0.26; proton group: 45.6% versus 52.6%, Chi² *p* = 0.73). In photon treated patients with concomitant and/or adjuvant CTx, we found a significantly higher pCEL incidence than in those with no or only neoadjuvant CTx (21.1% versus 4.3%, Chi² *p* = 0.02; for details see Supplementary Table 1).

10 patients (*n* = 4.1% of all patients and 5.6% of 178 CEL patients) were categorized as possible pCEL, including 8 patients fulfilling the criteria for definite or probable tumor progression in other localization.

### Tumor progression

In total, 132 patients (53.9%) had imaging findings indicating tumor progression (Table 2). Histological tissue examination was available in 88 of these patients (88/132 = 66.7%). In 5 of these patients, tissue examination revealed postradiogenic tissue injury in addition to tumor. 6 additional patients fulfilled the criteria for probable or possible pCEL in other localization. 44 patients fulfilled the imaging criteria of probable progressive disease (18%), 24 of them additionally presented with imaging findings indicative of pCEL in other localization. The incidence of definite or probable tumor progression was significantly higher in the photon therapy group (64.5%, *n* = 91/141) compared to the proton group (41.4%, *n* = 41/99) (*p* = 0.001). For the subgroup of HGG with available information regarding IDH status, tumor progression was more frequently observed in IDH-wildtype (76.5%, *n* = 95/124) versus IDH-mutant patients (23.9%, *n* = 17/71; Chi² *p* < 0.001).

In 36 cases (27.3%) with probable or definite progressive disease a methionine PET/MRI was performed. In 35/36 cases (97.2%) the respective CEL showed an increased amino acid metabolism. In one histological proven case of a progressive WHO grade III anaplastic astrocytoma the amino acid metabolism was not increased.

**Table 2:** Incidence of new or progressive contrast-enhancing lesions for the entire study population, the photon therapy and the proton therapy patient group

		Entire population (%)	Photon therapy (%)	Proton therapy (%)	*p*-value (Photon vs. Proton, Chi²)
Glioma	*n*	*n* = 240 (100)	*n* = 141 (58.8)	*n* = 99 (41.3)	
	Any new CEL	178 (74.2)	102 (72.3)	76 (76.8)	p >0.05
	Definite or probable radiation-induced CEL*	69 (28.8)	22 (15.6)	47 (47.5)	**p < 0.001**
	Definite or probable progressive disease**	132 (55)	91 (64.5)	41 (41.4)	**p < 0.05**
LGG	n	*n* = 25 (100)	*n* = 8 (32)	*n* = 17 (68)	
	Definite or probable radiation-induced CEL*	10 (40)	2 (25)	8 (47.1)	*p* > 0.05
	Definite or probable progressive disease**	4 (16)	4 (50)	0 (0)	*p* < 0.05
HGG	n	*n* = 215 (100)	*n* = 133 (61.9)	*n* = 82 (38.1)	
	Definite or probable radiation-induced CEL*	59 (27.4)	20 (15)	39 (47.6)	*p* < 0.001
	Definite or probable progressive disease**	128 (59.5)	87 (65.4)	41 (50)	*p* < 0.05

*CEL* contrast-enhancing lesion, *LGG* low grade glioma, *HGG* high grade glioma

*: including 5 cases with additional histological evidence of tumor tissue and 22 cases with additional imaging findings indicative of progressive disease in other localization.

**: including 5 cases with additional evidence of postradiogenic tissue injury on histological tissue examination and 28 cases with additional imaging findings indicative of possible or probable postradiogenic CEL in other localization.

^1^Histological tissue examination available, demonstrating radiation-induced tissue injury, including 5 cases with additional histological evidence of tumor tissue and 2 patients with additional imaging findings indicative of progressive disease in other localization.

^2^Patients fulfilling the imaging criteria for probable pCEL, including 20 cases with additional imaging findings indicative of progressive disease in other localization, no tissue examination performed.

^3^Patients fulfilling the imaging criteria for possible pCEL, including 8 cases with additional imaging findings indicative of progressive disease in other localization, no tissue examination performed.

^4^Patients with new CEL after RT not fulfilling the imaging criteria for possible or probable pCEL, including 24 cases with additional imaging findings indicative of pCEL other localization, no tissue examination performed.

^5^Histological tissue examination available, demonstrating vital tumor cells, including 5 cases with additional histological evidence of radiation-induced tissue injury and 6 cases with additional imaging findings indicative of pCEL in other localization.

#### Time interval from RT to occurrence of new or progressive CEL

**Fig. 5:**
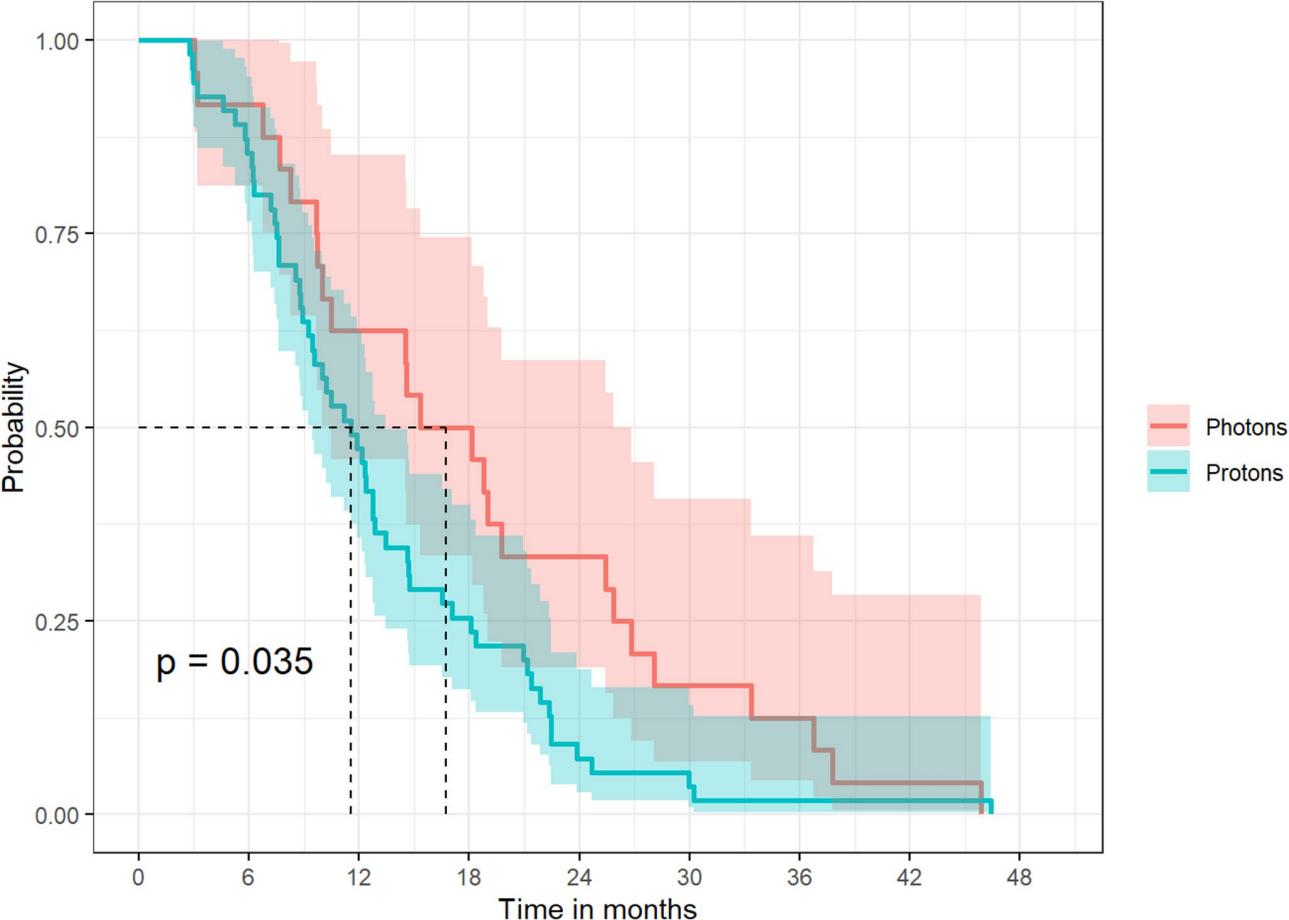
Kaplan-Meier plot showing the time interval between end of radiotherapy and first evidence of a new postradiogenic contrast-enhancing lesion (pCEL) for all patients with any pCEL during follow-up. The time after radiotherapy is shown in months on the x-axis. The y-axis describes the chance of survival without any pCEL. 1.00 represents 100% of the respective cohort with any pCEL. In patients treated with protons (turquoise curve) pCELs were observed significantly earlier than after photon therapy (red curve)

The median time interval between end of RT and first evidence of pCEL of any category was 16.5 months (range 3–45.3.3 months) after photon therapy and 11.4 months (range 2.7–45.8 months) after proton therapy (*p* = 0.035) (Fig. 5). For the subgroup of definite or probable pCEL, the median time interval was 14.8 months (range 3–45.3.3 months) after photon therapy and 11 months (range 2.7–45.8 months) after proton therapy (*p* = 0.061).

Tumor progression-related CELs occurred at a median time interval of 17 months (range 1.1–81.1 months) in the photon group, and 15.2 months (range 2.7–62.7 months) in the proton group, respectively. The difference was not significant (*p* = 0.98).

## Discussion

### Definition of postradiogenic CEL

New or progressive contrast-enhancing lesions on MRI after radiotherapy which are not related to disease progression, but represent posttherapeutic changes, have initially been described as “pseudoprogression” in high grade gliomas [23, 24]. However, pCEL do not describe a uniform entity, but rather constitute an umbrella term for any new parenchymal contrast-enhancing lesions due to long-term side effects of radiation therapy [6]. These include radiation necrosis as well as other lesions variously described in literature as “pseudoprogression”, “blood-brain barrier disruptions” (BBD), “speckled contrast enhancing lesions” (SCEs), and/or “radiation induced-contrast enhancements” (RIBI) [6, 15, 25] for example. Unfortunately, the exact definition of the respective phenomena differs significantly between studies in existing literature, which hampers comparison between studies [6]. To account for this lack of precise definition of the different forms of postradiogenic changes and their non-uniform imaging presentation, ranging from non-measurable, dot-like or speckled lesions to large, tumor-like, progressive, centrally necrotic lesions (typical “radiation necrosis”), we included any new or progressive contrast-enhancing lesion in our analysis and defined different categories of diagnostic certainty regarding their postradiogenic etiology. For definite diagnosis of radiation-induced CEL versus tumor-progression related CEL histological tissue examination was required, while probable diagnosis was based on imaging presentation with spontaneous lesion regression during available follow-up. We only assessed CELs occurring 3 months or later after the end of radiation therapy, therefore classical, early “pseudoprogression” lesions were not analyzed in our study. This has to be taken into account if comparing our results with existing literature.

### Incidence of postradiogenic CELs

*In our cohort of adult glioma patients and intracranial high dose RT we found any new or progressive CEL in 74.2% and definite*,* probable or possible pCEL in 32.9% of cases during a mean follow-up period of 30 months after RT. The incidence of probable or definite pCEL both in the total study population and in the subgroup of patients with high grade glioma (HGG) was significantly higher after proton versus photon therapy (47.5% and 47.6% for protons versus 15.6% and 15% for photons).*

*While the incidence of “classical”*,* early pseudoprogression*,* occurring within the first 90 days after radiation therapy*,* has been reported to be significantly lower in secondary (IDH mutant) glioblastoma*,* compared to wild-type glioblastoma* [26, 27], *we observed no significant difference in pCEL incidence in IDH-mutant versus IDH wildtype HGG in our analysis*,* which only assessed CELs occurring beyond the time period of classical pseudoprogression. However*,* as our paper was not specifically designed to assess the influence of IDH mutation status on postradiogenic changes and taking into account*,* that the percentage of IDH mutant versus IDH wildtype cases differed between therapeutic groups*,* these results have to be interpreted with caution.*

For the small subgroup of low-grade glioma (LGG), we also observed a higher incidence of pCEL in the proton (47.1%) compared to the photon group (25%), yet this difference was not significant. Probable or definite tumor-progression-related CEL were more common in photon patients (64.5%) than in patients treated with protons (41.4%). Postradiogenic CEL occurred earlier after proton therapy compared to photon therapy, while there was no significant difference regarding time interval between RT and first detection of tumor-progression-related CEL between the two groups.

### Postradiogenic CELs after photon therapy

After photon therapy, we observed an incidence of definite or probable pCEL of 15.2% for all glioma patients. In the high-grade glioma group, definite or probable pCELs occurred in 14.8% of patients, and in the small group of patients with low-grade gliomas, they were found in 20% of cases.

According to a review published in 2008, the reported incidence rates of radionecrosis after photon therapy for brain tumors ranged from 3 to 24%, while pseudoprogression occured in 9–31% of patients after standard temozolomide chemoradiotherapy [28]. A more recent meta-analysis including 73 studies on 3781 adult patients with WHO °III (7%) r °IV (89%) lioma found any new or progressive contrast enhancement during a mean follow-up period of 14 months (range 1–67 months) in 68.8% o patients after standard of care treatment [29]. The etiology of these CEL was determined based on follow-up imaging in 75% of caes, on histological tissue examination in 20%, on acombination of both in 2%, and n clinical follow-up alone in 1% of caes. CEL were categorized as postradiogenic in 36%, tumo progression-related in 60%, and emained unclear in 4% of caes. In our cohort, we observed pCELs after photon therapy only in 15.9% of caes. One potential explanation for the higher incidence reported in the meta-analysis compared to our results, might be the inclusion of patients with “classical” pseudoprogression which occurs typically within three months after end of therapy [6, 30], in this meta-analysis. In our institution, we do not perform any regular follow-up MRI within the first three months after end of RT, and therefore classical pseudoprogression should be underrepresented in our cohort. The mean time interval between the end of the concomitant chemoradiotherapy and evidence of new or progressive lesions was only available in a small subset of 11 studies included in the meta-analysis. It was 13.0 months for treatment related changes (comparable to 14.8 months in our cohort) and 10.5 months for tumor progression-related changes (15.2 months in our photon patient group) [29].

A recent two-center study on patients with IDH mutated grade 2 glioma found a cumulative incidence of new treatment-related CELs of 13% within the first year, 22% after 5 years and 28% after 10 years of follow-up [31]. In accordance to our study, the authors defined those lesions as proposed by van West et al. as new contrast enhancing lesion vanishing or remaining during follow-up, or remaining stable without treatment over a period of at least 12 months [32]. The incidence rates in this grade 2 cohort was very similar to the incidence rate in our cohort comprising predominately high-grade gliomas.

More recently developed, arc-based rotation photon techniques, as Volumetric-modulated arc-therapy and helical tomotherapy can achieve highly conformal dose distributions with improved target volume coverage, which might reduce side-effects by sparing normal-appearing brain tissue compared to the conventional photon techniques used in our cohort [33]. The potential impact of those new methods on the incidence of pCELs should be subject of further investigations.

### Postradiogenic CELs after proton therapy

The incidence of definite or probable pCEL after proton therapy was 47.5% in our whole study population, 47.6% in the high-grade glioma patients, and 47.1% in the smaller subgroup of low-grade gliomas.

The existing literature on proton therapy-related CEL predominately focuses on pediatric cases and/or on low grade tumors [12, 15–17, 34–36]. A study on 194 adult patients with IDH mutant WHO grade 2 (128, 66%) and grade 3 glioma (66, 34%) found adiation induced-contrast enhncement (referred to as “RICE” and defined as new, treatment-related CEL in this publication) within the 80% isodose line in a total of 25% of caes, in 29% of WHO grade 2 glioas, and in 17% o WHO grade 3 tumors during a medianfollow-up period of 5.1 years. The authors found an increased risk of developing these treatment-related new CELs with older age, independent of tumor characteristics. The lesions occurred at a median time interval of 16 months after treatment (range; 2–41 months) [37]. The reported CEL incidence was markedly lower compared to our cohort, which might be explained by the fact, that only lesions occurring within the 80% isodose were considered.

A previous study, performed at our own institution on 42 adult patients with newly diagnosed WHO grade II or III glioma observed pCEL (referred to as “RIBI” in this study) in 21 (50%) of cases [25]. Here, any new CEL distant from the surgical defect was included, comparable to our current study.

In an analysis of 99 adult patients with low-grade glioma, no significant difference was found between photons (14.3%) and protons (16%) with regard to the incidence of so-called pseudoprogression within 6 months after irradiation. However, the authors did not consider postradiogenic changes that occurred later, so comparability with our study is limited [18].

In another study with 111 pediatric patients, no difference was found after proton therapy for medulloblastoma compared to cohorts with photon therapy. The low rate of CNS injury of 3.6% after 5 years can be explained, among other things, by the fact that only clinically symptomatic MRI changes were assessed [35].

A meta-analysis including five pediatric and 3 adult cohort studies with 517 pediatric and 424 adult low grade glioma patients found similar rates of pCELs (referred to as “pseudoprogression” in this paper) in the pediatric cohorts after proton (33%; 95%CI, 20–47%) and photon therapy (34%; 95%CI, 23–45%). In the adult cohorts however, the incidence was significantly higher after proton (30%; 9 5% CI, 21 − 3 9%) compared to photon therapy (18%: 95% CI, 12–25%) [16] which is in accordance with our observations. Interestingly, in our adult cohort with predominately high-grade glioma, the incidence of definite or probable pCEL after proton therapy was even higher (47%) han found in this meta-analysis. It has to been taken into account that the definition of the term pseudoprogression in the individual studies included in the meta-analysis was very heterogeneous.

As mentioned above, several factors might account for the reported differences in literature regarding the incidence of pCELs after both proton and photon therapy. Besides the different patient populations analyzed in the studies, in particular the varying definitions of pCELs regarding their morphology, as well as their time of occurrence and localization with respect to the radiation field play an important role in this regard. In addition, different radiation methods, as e.g. dual-scattering versus pencil-beam scanning proton therapy might also account for varying pCEL incidences between studies. In our cohort we did not observe any significant differences between those two proton therapy approaches, however, the majority of our patients has been treated with dual-scattering proton therapy, while pencil-beam scanning has only been applied in 20% of cases.

### Postradiogenic CELs and chemotherapy

In accordance with the literature [38] we observed a significantly higher incidence of pCEL in our photon-treated patients with concomitant and/or adjuvant CTx compared to those without. For low grade glioma, a higher risk (HR = 2.1) of pCELs (named “pseudoprogression” in the respective paper) has been described after proton therapy and adjuvant CTx, while CTx risk differed not between patients with or without concomitant ± adjuvant CTx [39]. In our proton cohort, with predominately high-grade glioma (82.8%) and >50% IDH wildtype glioblastoma, we didn´t find any difference regarding pCEL incidence neither for patients with adjuvant nor for those with concomitant +/- adjuvant CTX compared to cases with no or only neoadjuvant CTx.

## Reasons for higher incidence and earlier appearance of pCEL after proton versus photon therapy

In our cohort, we found significantly more pCEL in patients treated with protons than in patients treated with photons. The pCELs also occurred earlier after proton compared to photon therapy. One possible explanation could lie in the variable biological effectiveness (RBE) of protons. In radiation planning, a constant RBE of 1.1 has been assumed in comparison to X-rays [40]. However, several studies discuss a variable RBE, which can be higher than 1.1 depending on the position relative to the Bragg peak [12, 14, 25]. Due to the location of many primary brain tumors at the level of the lateral ventricles, the distal radiation field often comprises the periventricular region, an area of assumed increased radiosensitivity and increased clinical RBE [10, 25]. This, in combination with the overall high dose irradiation of 54–60 Gy, close to the tolerance limit of healthy brain tissue [13], could be a reason for the higher incidence after proton therapy.

### CEL localization

There is evidence that CEL after proton therapy do not occur randomly, but predominately develop at sites of increased linear energy transfer (LET) and in vicinity to the ventricular system [10, 25]. In a study on 110 patients with low grade glioma who received proton therapy, 90% of the ELs were located at the distal edge of at least one beam, 69% of themoccurred within 4 mm distance and 92% within 0 mm distance from the ventricular system. A model for patient-level risk prediction based on these data predicted a three-fold increased risk within 4 mm around the ventricles [10].

### Local tumor control

Based on methodological considerations, the advantage of proton over photon therapy is based on reduction of treatment related side-effects, rather than in improvement of local tumor control [2–4]. In our cohort, we observed a lower incidence of tumor-progression-related CEL after proton versus photon therapy (41.4% versus 64. %). However, our study was not designed to compare the effectiveness of proton versus photon therapy with regard to prevention of tumor growth or tumor recurrence. In particular, the photon therapy group comprised significantly more patients with WHO grade IV tumors (glioblastoma) than WHO grade III cases, while those histological subgroups were equally represented in the proton therapy group. Therefore, these findings have to be interpreted with caution. Yet, they are in accordance with a national cancer data-based analysis, which found a superior median and 5 year survival in glioma patients after proton therapy compared to photon therapy (170 proton patients, almost 50.000 photon patients, 91.2% high gradegliomas, median age 59 years) [41].

### Strengths and limitations of the study

The strengths of our evaluations are the relatively large patient cohort, the standardized follow-up and the precise definition of postradiogenic versus tumor-progression-related CELs at different levels of diagnostic certainty.

Considerable limitations are given by the retrospective design of the study and the fact that histological tissue examination was only available in 43% of cases, and therefore the diagnosis of a definite postradiogenic etiology could only be made in a relatively small subset of patients. In addition, it has to be considered that we included all new or progressive CELs lesions independent of their clinical relevance (asymptomatic and symptomatic cases) and independent of type of concomitant medical treatment.

As we included only patients treated before 2020, tumor diagnoses in our cohort were made based on the WHO 2016 classification. As the therapeutic management of the individual cases had been chosen based on these initial diagnoses, we decided to use those initial diagnosis for statistical analysis. Re-classification according to the latest 2021 WHO classification – if possible - resulted in an upgrade from low- to high-grade glioma in three cases, and therefore pCEL incidence in LGG versus HGG might differ slightly, if treatment-decisive diagnosis had been made based on the 2021 classification.

Median age of proton patients was slightly lower than that of the photon group, and the proton group comprised a higher proportion of LGG patients as well as a higher proportion of IDH-mutant HGG compared to the photon group. This was not due to systematic assignment of specific patient groups to one of both treatment options, but might rather be explained by the fact that younger patients, which more frequently suffer from IDH mutant gliomas, are more prone to inform themselves about treatment alternatives and to contact a proton center despite long travel distances.

A subset of follow-up MRI-scans were carried out in an outpatient setting on scanners from different manufacturers with different field strengths, which may limit comparability in individual cases.

## Conclusion

In our retrospective study of 240 adult patients with gliomas treated with high-dose irradiation, we found an overall high incidence of postradiogenic CELs. These posttherapeutic changes were more frequently observed and occurred earlier after proton versus photon therapy.

The correct interpretation and management of these findings particularly after proton, but also after photon therapy, represent a diagnostic challenge for the radiologist in clinical routine. Awareness of their highly variable MR morphology and their time course is essential for correct interpretation and management, particularly in the setting of the heterogeneous and inconsistent definitions of postradiogenic tissue effects in existing literature.

## Supplementary Information

Below is the link to the electronic supplementary material.

Supplementary Material 1 (DOCX 19.5 KB)

## Data Availability

The clinical MR data sets analyzed in this study are not openly available due to reasons of sensitivity and are available from the corresponding author upon reasonable request. Data are located in controlled access data storage at University Hospital Dresden.
