# Supplementary material for: Incidence and time course of new contrast-enhancing lesions on MRI after proton versus photon radiotherapy in glioma patients
Source: Neuroradiology. 2025 Nov 24;68(2):517–31. doi: 10.1007/s00234-025-03829-1 (PMC13021816; doi:10.1007/s00234-025-03829-1)
Supplement: Supplementary file 1 — Supplementary Material 1 (DOCX 19.5 KB) [file 234_2025_3829_MOESM1_ESM.docx]

**Supplementary Table 1**: Incidence of probable or definite radiation-induced contrast-enhancing lesions in patients with or without chemotherapy

| **Radiation therapy**  n (%) | **Type of CTX (all*)**  n (%) | | **pCEL+**  n (%) | **pCEL-**  n (%) | **p-value (Chi²)** |
| --- | --- | --- | --- | --- | --- |
| **Whole population**  240 (100) | **No or only neoadjuvant CTX**  77 (32.1) | | 18 (23.4) | 59 (76.6) | p > 0.05 |
|  | **Concomitant and/or adjuvant CTX**  163 (67.9) | | 51 (31.3) | 112 (68.7) |  |
| **Photon therapy**  141 (100) | **No or only neoadjuvant CTX**  46 (32.6) | | 2 (4.3) | 44 (95.7) | **p < 0.05** |
|  | **Concomitant and/or adjuvant CTX**  95 (67.4) | | 20 (21.1) | 75 (78.9) |  |
|  |  | **Only adjuvant CTX**  19 (32.6) | 4 (21.1) | 15 (78.9) | p > 0.05^1^ |
|  |  | **Concomitant ± adjuvant CTX**  76 (53.9) | 14 (18.4) | 62 (81.6) | p > 0.05^2^ |
| **Proton therapy**  99 (100) | **No or only neoadjuvant CTX**  31 (31.3) | | 16 (52.6) | 15 (48.4) | p > 0.05 |
|  | **Concomitant and/or adjuvant CTX**  68 (68.7) | | 31 (45.6) | 37 (54.4) |  |
|  |  | **Only adjuvant CTX**  36 (36.4) | 20 (55.6) | 16 (44.4) | p > 0.05^1^ |
|  |  | **Concomitant ± adjuvant CTX**  32 (32.3) | 13 (40.6) | 19 (59.4) | p > 0.05^2^ |

CTX: chemotherapy; pCEL: definite or probable postradiogenic contrast-enhancing lesions; * “All” refers to number (percentage) of patients with and without definite or probable postradiogenic contrast-enhancing lesions; pCEL+: patients with definite or probable postradiogenic contrast-enhancing lesions; **: “No CTX” indicates no or only neoadjuvant CTX:

1: only adjuvant versus no or only neoadjuvant CTX; 2: concomitant ± adjuvant CTX versus no or only neoadjuvant CTX

| **HGG with available IDH status**  n (%) | **HGG, IDH+**  n (%) | **HGG, IDH-**  n (%) | **p-value (Chi²)** |
| --- | --- | --- | --- |
| **Whole population**  195 (100) | 71 (36.4) | 124 (63.6) |  |
| **Photon therapy group**  112 (100) | 31 (27.7) | 81 (72.3) | **p < 0.05** |
| **Proton therapy group**  83 (100) | 40 (48.2) | 43 (51.8) |  |

**Supplementary table 2a:** High grade glioma IDH-mutation status in photon versus proton therapy groups

HGG: high grade glioma; IDH: IDH-mutant; IDH-: IDH wildtype

**Supplementary table 2b:** Incidence of definite or probable radiation-induced CEL after photon and proton therapy for IDH-wildtype versus IDH-mutant high-grade glioma

| **HGG with available IDH status**  n (%) | | **HGG, IDH+**  n (%) | **HGG, IDH-**  n (%) | **p-value (Chi²)** |
| --- | --- | --- | --- | --- |
| **Whole population**  195 (100) | Whole population, **CEL+**  32 (16.4) | 24 (12.3) | 27 (13.8) | p > 0.05 |
|  | Whole population, **CEL-**  163 (83.6) | 47 (24.1) | 97 (49.7) |  |
| **Photon therapy group**  112 (100) | Photon therapy; **CEL+**  13 (11.6) | 1 (0.01) | 12 (10.7) | p > 0.05 |
|  | Photon therapy; **CEL-**  99 (88.4) | 30 (26.8) | 69 (61.6) |  |
| **Proton therapy group**  83 (100) | Proton therapy; **CEL+**  38 (45.8) | 23 (27.7) | 15 (18.1) | p > 0.05 |
|  | Proton therapy; **CEL-**  45 (54.2) | 17 (20.5) | 28 (33.7) |  |

HGG: high grade glioma; IDH: IDH-mutant; IDH-: IDH wildtype; pCEL+: patients with definite or probable postradiogenic contrast-enhancing lesions; pCEL-: patients without postradiogenic contrast-enhancing lesions
